# Post-Infectious Acute Transverse Myelitis in a COVID-19 Patient

**DOI:** 10.7759/cureus.98649

**Published:** 2025-12-07

**Authors:** Jonathan Fabricante, Sophie Cooper, Lal Kumar, Azeem Alam

**Affiliations:** 1 Internal Medicine, The Princess Alexandra Hospital NHS Trust, Harlow, GBR; 2 Clinical Radiology, Imperial College London, London, GBR

**Keywords:** acute transverse myelitis (atm), covid 19, para-infectious covid myelitis, post-covid-19 sequelae, tranverse myelitis

## Abstract

A 41-year-old man with no comorbidities presented with features consistent with a subacute partial upper thoracic myelopathy, two days after treatment in the emergency department for acute urinary retention. On examination, he was ambulatory but had an unsteady gait and lower limb weakness, with brisk reflexes, upgoing plantars, and subtle sensory changes at the T4-T5 level. The patient developed neurological symptoms eight days after the initial onset of COVID-19. An urgent magnetic resonance imaging (MRI) of the spine demonstrated a T2 hyperintense signal extending from C3 to the conus, while cerebrospinal fluid analysis revealed lymphocytosis. Following treatment with high-dose intravenous methylprednisolone, the patient showed significant clinical improvement, with recovery of strength and bladder and bowel function. This case highlights COVID-19 as a possible post-infectious trigger for transverse myelitis and underscores the need to investigate acute urinary retention in young patients.

## Introduction

Following the emergence of coronavirus disease 2019 (COVID-19), caused by severe acute respiratory syndrome coronavirus 2 (SARS-CoV-2), an expanding range of systemic complications has been increasingly recognized. Although respiratory manifestations predominate, neurological complications, including anosmia, encephalopathy, stroke, and demyelinating syndromes, have also been reported [1,2]. Among these, acute transverse myelitis (ATM) is a rare but serious condition characterized by spinal cord inflammation leading to varying degrees of motor, sensory, and autonomic dysfunction [3]. Incidence of ATM is between 1.34-4.6 per million cases per year with bimodal peaks between ages 10-19 and 30-39 years old, with no gender or familial or ethnic predisposition [4].

In the context of COVID-19, ATM is hypothesized to result from either direct viral neuroinvasion, supported by occasional detection of SARS-CoV-2 RNA in cerebrospinal fluid, or a post-infectious immune-mediated mechanism, suggested by the delayed onset of neurological symptoms and favorable response to immunotherapy [5,6]. In a 2021 case report by Roman et al., 43 patients with COVID-19-associated ATM from 21 countries were documented between March 2020 and January 2021, highlighting the global occurrence yet relative rarity of this complication [7]. 

Early recognition and prompt treatment with high-dose corticosteroids are crucial to optimize outcomes [7]. We present a case of a previously healthy 41-year-old man who developed subacute partial thoracic myelopathy with urinary retention shortly after symptomatic COVID-19 infection. This case highlights the importance of considering post-infectious neurological complications following COVID-19 and investigating acute urinary retention in younger patients for possible central causes.

## Case presentation

A 41-year-old previously healthy man with no comorbidities presented to the Emergency Department (ED) with 24 hours of acute urinary retention. Six days earlier, he had self-tested positive for COVID-19 and experienced fever and generalized flu-like symptoms without respiratory distress. He denied any limb weakness or sensory changes at that time, and no formal neurological examination was performed. There was no history of lower urinary tract symptoms, although a prostate examination was not performed. He was discharged with an outpatient trial-without-catheter appointment.

On day 8 of his COVID-19 illness, two days after his initial ED visit, he developed numbness over his torso, progressive weakness in both lower limbs, and constipation. He remained pyrexic (38-39.5°C), although a repeat COVID-19 test was now negative. He denied back pain but described a warm, spreading sensation from his torso to his lower limbs and constipation for three days.

On examination, findings were consistent with a partial upper thoracic myelopathy. He was ambulatory but had an unsteady gait, with muscle power graded 3/5 in knee and hip flexion and 4/5 in ankle extension and flexion. Reflexes were brisk with clonus and bilateral upgoing plantars. Sensory examination revealed subtle impairment to the T4-T5 level, while upper limb and cranial nerve examinations were normal.

He was admitted for urgent magnetic resonance imaging (MRI) of the spine and neurology review to investigate possible post-infectious transverse myelitis (TM). The initial standard blood panel was unremarkable, with a C-reactive protein (CRP) level of 5 mg/L, and no lymphopenia was observed. His Vitamin B12, folate, thyroid-stimulating hormone (TSH), HbA1c, urea and electrolytes, and liver function tests were all in normal range (Table 1).

**Table 1:** Initial blood tests showing all parameters within normal limits WBC: Whole blood cells, CRP: C-reactive protien; eGFR: TSH: Thyroid-stimulating hormone, eGFR: Estimated glomerular filtration rate

Parameter	Result	Reference Range
WBC	10.8 x10^9^/L	4 - 11 x10^9^/L
Neutrophils	7.3 x10^9^/L	2 -8 x10^9^/L
Lymphocytes	2.7 x10^9^/L	1 - 4.8 x10^9^/L
CRP	5 mg/L	<5 mg/L
Vitamin B12	561 ng/L	180 - 914 ng/L
TSH	0.60 mU/L	0.35 - 5.00 mU/L
Folate	7.6 ug/L	2.7 - 34.0 ug/L
Creatinine	99 umol/L	53 - 97 umol/L
eGFR	>60	60 - 150
Urea	5.7 mmol/L	2.5-7.8 mmol/L
Sodium	139 mmol/L	133 - 146 mmol/L
Potassium	4.5 mmol/L	3.5 - 5.3 mmol/L

**Figure 1:**
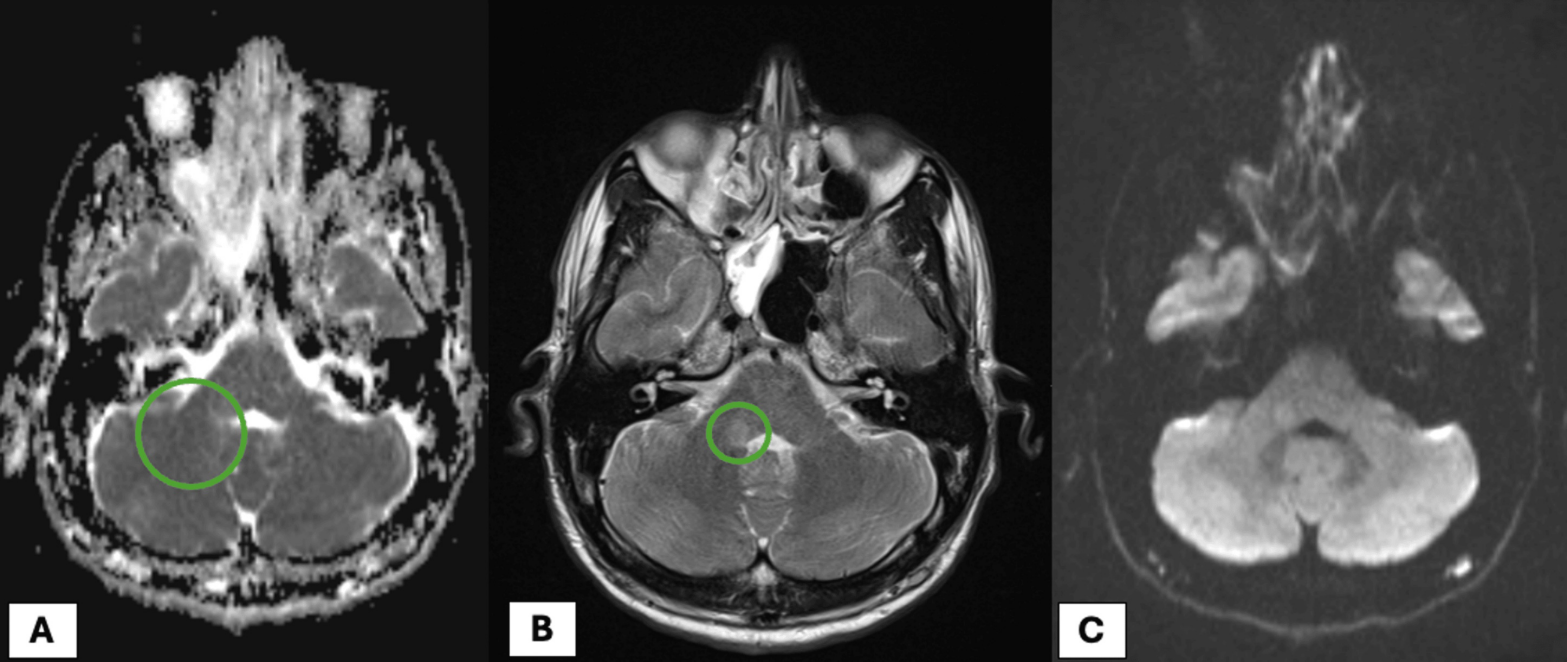
MRI head showing a focal T2 hyperintensity in the right posterior fossa involving the middle cerebellar peduncle, extending into the medulla and abutting the fourth ventricle. There is no associated enhancement or restricted diffusion. Findings are consistent with an inflammatory process.

An MRI of the head showed a focal T2 hyperintensity in the posterior fossa on the right side involving the middle cerebellar peduncle extending into the medulla abutting the fourth ventricle. A mild T2 shine-through was noted on the DWI sequence (Figure 1).

**Figure 2:**
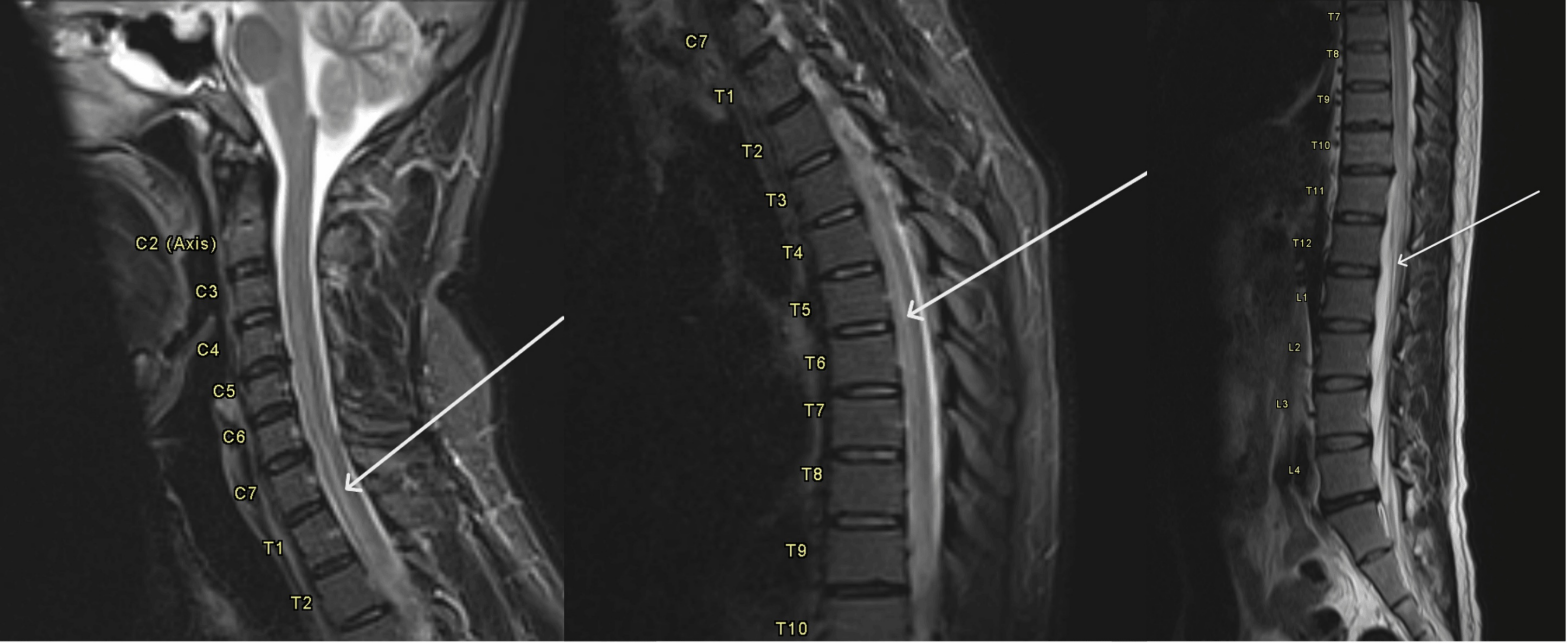
MRI spine showing an inhomogeneous central T2 hyperintense cord signal abnormality extending from C3 to the conus with no associated enhancement, consistent with inflammatory myelopathy.

An MRI of the whole spine showed an inhomogeneous, centrally T2-hyperintense cord signal abnormality extending from the C3 level to almost the conus. No contrast enhancement was observed, and only minimal diffuse spinal cord swelling was noted (Figure 2).

With diffuse central cord signal abnormality and associated focal brain lesion (right middle cerebellar peduncle and medulla), occurring in the setting of a previous viral infection, MRI findings were compatible with acute disseminated encephalomyelitis (ADEM).

Cerebrospinal fluid (CSF) obtained by lumbar puncture demonstrated elevated glucose levels with a normal protein concentration of 0.38 g/L.CSF analysis revealed a predominance of lymphocytes, with no organisms detected on Gram stain and no growth observed on prolonged culture (Table 2).

**Table 2:** Cerebrospinal fluid (CSF) analysis showed mild lymphocytic pleocytosis

Parameter	Result	Reference Range
CSF Biochemistry		
CSF glucose	5.6mmol/L	2.5-4.5
Serum glucose	8.3mmol/L	3.0 - 7.7
CSF protein	0.38g/L	0.15-0.45
CSF cell count and microscopy		
CSF polymorphs	2%	-
CSF lymphocytes	99%	-
Cell count	60/mm^3^	-
Gram stain	No organisms seen	-
Culture result	No growth after prolonged incubation	-
Molecular amplification test for Mycobacterium tuberculosis complex	Negative	-

Autoimmune serology revealed a positive antinuclear antibody (ANA) test, with negative anti-double-stranded DNA antibodies. The rest of the extended panel (anti-CENP, extractable nuclear antigen (ENA), anti-Ro, anti-La, anti-Sm, anti-Jo 1, anti-Scl7 0, anti-U1RNP, anti-neutrophil cytoplasmic antibodies (ANCA)) was negative. Anti-aquaporin 4 and Anti-Myelin Oligodendrocyte Glycoprotein (Anti-MOG) were also noted to be negative. Oligoclonal IgG was present in the CSF with no corresponding abnormality in the serum. Serology for hepatitis B, C, E, HIV, syphilis, and *Borrelia* was negative.

The clinical picture of subacute upper motor neuron signs required urgent MRI of the brain and spinal cord. Given his subacute progression, a vascular or compressive myelopathy was unlikely. The patient's history disclosed no alcohol use, nutritional deficits, or other exposures that would raise concern for a metabolic aetiology. Given the absence of prior episodes and the MRI and CSF findings, multiple sclerosis was deemed unlikely. Given the MRI findings described above, the absence of encephalopathy or altered mental status, and the progressive onset of neurological symptoms following a viral infection, acute transverse myelitis (ATM) was considered far more likely than acute disseminated encephalomyelitis (ADEM) as the cause of the patient’s presentation.

The patient was started on methylprednisolone 500mg intravenously for a total of 7 days, which he was able to complete in an outpatient setting. This was transitioned to oral prednisolone 60 mg daily for one week, followed by a tapering regimen over two weeks. The patient was also commenced on intravenous acyclovir; however, this was discontinued after two days as the presentation was felt to represent a post-viral sequela rather than an active viral infection.

On completion of the IV methylprednisolone, his symptoms improved, and he had 5/5 muscle power on all extremities, bowel control had returned, and he was able to mobilise independently. After completion of two weeks of prednisolone, the patient was able to void freely and was back to his functional baseline. A repeat MRI of the head and spine one month after presentation showed no evidence of progressive disease.

## Discussion

COVID-19 has been associated with a broad spectrum of clinical manifestations, ranging from mild respiratory illness to severe multi-organ involvement. Neurological complications, though less common, are increasingly recognized and include encephalopathy, cerebrovascular events, and immune-mediated disorders such as ATM [8].

In the present case, the temporal relationship between SARS-CoV-2 infection and the onset of neurological symptoms supports an immune-mediated mechanism rather than direct viral invasion. ATM involves inflammation across the spinal cord, resulting in motor, sensory, and autonomic dysfunction below the level of the lesion [8,9]. Post-infectious TM is thought to occur when an aberrant immune response, triggered by viral antigens, cross-reacts with host myelin proteins, leading to demyelination and neuronal injury. COVID-19-related TM may result from a similar process, where molecular mimicry and cytokine-mediated inflammation (particularly elevated IL-6 and TNF-α levels) contribute to spinal cord damage.

Our patient developed lower limb weakness, sensory changes, and urinary retention eight days after COVID-19 infection, consistent with a post-infectious immune-mediated etiology. MRI findings and the patient’s favorable response to high-dose methylprednisolone further support an inflammatory process. Comparable cases have been documented in the literature. For example, a 60-year-old man developed limb weakness and urinary retention 10 days post-COVID-19 infection and demonstrated clinical improvement after corticosteroid therapy [10].

High-dose corticosteroids remain the cornerstone of ATM management, with plasma exchange reserved for refractory cases [11]. Early recognition and prompt immunomodulatory therapy are crucial for optimizing recovery, as most patients achieve at least partial functional improvement with rehabilitation [12].

This case underscores COVID-19 as a potential post-infectious trigger for transverse myelitis and highlights the importance of considering this diagnosis in young patients presenting with acute urinary retention and lower limb weakness. A limitation of this report is the absence of viral PCR testing in cerebrospinal fluid, which prevents complete exclusion of direct viral neuroinvasion. Nonetheless, the clinical and temporal features strongly suggest a post-infectious immune-mediated mechanism.

## Conclusions

This case illustrates acute transverse myelitis as a potential post-infectious complication of COVID-19, even in previously healthy individuals. The patient’s clinical presentation, imaging, and cerebrospinal fluid findings were consistent with an inflammatory myelopathy likely triggered by a recent SARS-CoV-2 infection. Early recognition and prompt corticosteroid therapy led to full recovery. Clinicians should maintain a high index of suspicion for immune-mediated spinal cord disorders in patients presenting with new-onset myelopathy or unexplained urinary retention following viral illness.

## References

[1] Ellul MA, Benjamin L, Singh B (2020). Neurological associations of COVID-19. Lancet Neurol.

[2] Paterson RW, Brown RL, Benjamin L (2020). The emerging spectrum of COVID-19 neurology: clinical, radiological and laboratory findings. Brain.

[3] Transverse Myelitis Consortium Working Group (2002). Proposed diagnostic criteria and nosology of acute transverse myelitis. Neurology.

[4] Bhat A, Naguwa S, Cheema G, Gershwin ME (2010). The epidemiology of transverse myelitis. Autoimmun Rev.

[5] Munz M, Wessendorf S, Koretsis G (2020). Acute transverse myelitis after COVID-19 pneumonia. J Neurol.

[6] Jacob A, Weinshenker BG (2008). An approach to the diagnosis of acute transverse myelitis. Semin Neurol.

[7] Román GC, Gracia F, Torres A, Palacios A, Gracia K, Harris D (2021). Acute transverse myelitis (ATM): clinical review of 43 patients with COVID-19-associated ATM and 3 post-vaccination ATM serious adverse events with the ChAdOx1 nCoV-19 vaccine (AZD1222). Front Immunol.

[8] Liotta EM, Batra A, Clark JR, Shlobin NA, Hoffman SC, Orban ZS, Koralnik IJ (2020). Frequent neurologic manifestations and encephalopathy-associated morbidity in Covid-19 patients. Ann Clin Transl Neurol.

[9] Lopez Chiriboga S, Flanagan EP (2021). Myelitis and other autoimmune myelopathies. Continuum (Minneap Minn).

[10] Chow CC, Magnussen J, Ip J, Su Y (2020). Acute transverse myelitis in COVID-19 infection. BMJ Case Rep.

[11] Greenberg BM (2011). Treatment of acute transverse myelitis and its early complications. Continuum (Minneap Minn).

[12] Defresne P, Hollenberg H, Husson B (2003). Acute transverse myelitis in children: clinical course and prognostic factors. J Child Neurol.

